# Synthesis and Future Electronic Applications of Topological Nanomaterials

**DOI:** 10.3390/ijms25010400

**Published:** 2023-12-28

**Authors:** Gangtae Jin, Seo-Hyun Kim, Hyeuk-Jin Han

**Affiliations:** 1 Department of Materials Science and Engineering, Cornell University, Ithaca, NY 14853, USA; gj98@cornell.edu; 2 Department of Environment and Energy Engineering, Sungshin Women’s University, Seoul 01133, Republic of Korea; 220236120@sungshin.ac.kr

**Keywords:** topological materials, nanostructures, synthesis

## Abstract

Over the last ten years, the discovery of topological materials has opened up new areas in condensed matter physics. These materials are noted for their distinctive electronic properties, unlike conventional insulators and metals. This discovery has not only spurred new research areas but also offered innovative approaches to electronic device design. A key aspect of these materials is now that transforming them into nanostructures enhances the presence of surface or edge states, which are the key components for their unique electronic properties. In this review, we focus on recent synthesis methods, including vapor–liquid–solid (VLS) growth, chemical vapor deposition (CVD), and chemical conversion techniques. Moreover, the scaling down of topological nanomaterials has revealed new electronic and magnetic properties due to quantum confinement. This review covers their synthesis methods and the outcomes of topological nanomaterials and applications, including quantum computing, spintronics, and interconnects. Finally, we address the materials and synthesis challenges that need to be resolved prior to the practical application of topological nanomaterials in advanced electronic devices.

## 1. Introduction

To harness the potential of topological materials in electronics, it is essential to precisely manage and detect their states in nanostructures [1,2]. Nanostructures can amplify topological states due to their significantly large surface area-to-volume ratio, enhancing the contribution of the topological surface or edge states. This article delves into the synthesis of topological nanostructures ranging from topological insulators to semimetal/metal nanostructures of the Weyl and Dirac types and their application in electronics.

In the past decade, the field of condensed matter physics has seen the emergence of a novel class of materials known as topological materials [3]. These materials, characterized by their unique electronic band structures, diverge significantly from conventional insulators and metals [4,5,6]. Their distinct band topology gives rise to the robust, helical transport of the electrons, exhibiting a linear energy–momentum relationship at the material’s edge or surface. These materials exhibit topologically protected surface states that bring exotic electronic properties. In topological insulators (TIs), these surface states result from the inversion of the bulk bands due to the strong spin–orbit coupling and are protected by time reversal symmetry. The scope of topological materials has recently expanded to encompass topological semimetals (TSMs), notably Weyl and Dirac semimetals [7]. Weyl and Dirac semimetals are three-dimensional (3D) systems with gapless bulk states consisting of relativistic chiral fermions close to nodal points and Fermi arc surface states. The 3D Weyl and Dirac equations describe these systems, respectively. The early development of topological materials, including experimental and theoretical findings to TIs, and TSMs, have been extensively covered in several review articles [2,8,9,10,11,12].

Topologically protected surface states in materials provide attractive electronic properties for future advanced electronic applications. For example, the linear dispersion between momentum and energy in these materials is promising for high-speed electronics, as it allows for faster electron transport [13,14,15]. Additionally, the locking of spin and momentum in topological materials makes them ideal for spintronics, which involves the manipulation of electron spins for information storage and processing [16,17,18,19,20]. Furthermore, the directional motion of electrons in topological materials can enable the development of low-dissipation electronics, reducing energy loss and improving efficiency [21,22,23]. These advantages make topological materials highly attractive for advanced electronic applications. These distinct characteristics of the topological surface states have been extensively studied in bulk systems through surface-sensitive techniques; nevertheless, these properties have not been thoroughly studied in nanostructures [24,25,26,27,28].

Investigating the topological surface states within nanostructures offers a significant advantage, as the nanostructures enhance the role of the topological surface state transport and minimize the effect of bulk electronic states in transport measurements. Many TIs and TSMs have been successfully transformed into nanostructures. In early studies of layered TIs, Bi_2_Se_3_ and Bi_2_Te_3_ were achieved through direct synthesis or mechanical exfoliation. This transformation has broadened the horizons of quantum computing and spintronics. The reduced scaling of these materials allows unique quantum confinement effects [29,30], leading to new electronic and magnetic properties [18].

Among the various techniques mentioned, the vapor–liquid–solid (VLS) method is particularly favored for its ability to produce high-quality, single-crystalline nanostructures. On the other hand, chemical vapor deposition (CVD) offers scalability and versatility, making it suitable for large-scale production. Conversion methods, while less prevalent, provide an alternative route for obtaining topological nanomaterials from preexisting structures. The selection of a synthesis method is crucial, since it can significantly influence the quality, morphology, and properties of the resulting topological nanomaterials.

In this review, we introduce recent synthesis studies on nanostructures of TIs and Weyl and Dirac semimetals using VLS, CVD techniques, and conversion methods. We highlight the fabrication of topological nanomaterials that can lead to a paradigm shift in electronic technologies, such as quantum computing, advanced spintronics, and interconnects. Lastly, we address the remaining obstacles in fabricating topological nanostructures with high crystal quality and accurate morphological manipulation, along with the development of robust topological nanostructures.

## 2. Synthesis Methodologies of Topological Nanomaterials

**Figure 1:**
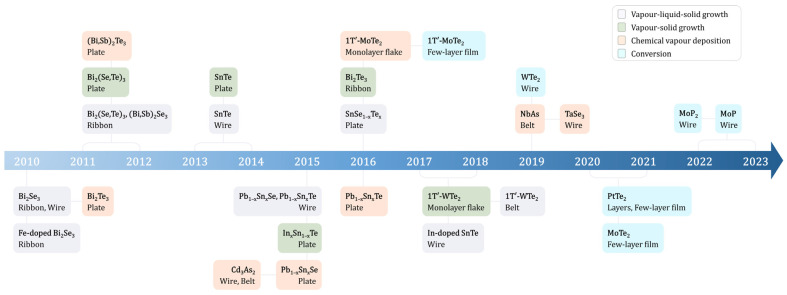
Timeline of the development of topological nanomaterials with various synthesis methods, such as vapor–liquid–solid, chemical vapor deposition growth, and conversion techniques.

Numerous TIs and TSMs have been transformed into nanostructures. While some are derived from bulk layered crystals via mechanical exfoliation, our focus here is on their direct synthesis. The VLS and CVD growth techniques are the predominant methods employed for crafting these nanostructures, especially for studying electrical transport attributes. Figure 1 provides a comprehensive timeline of the significant achievements in synthesizing nanomaterials, including topological insulators (TIs) and topological crystalline insulators (TCIs), as well as Weyl and Dirac semimetals.

In the realm of experimental demonstrations, the exploration of topological materials has predominantly centered around their bulk forms, achieved through specialized single-crystal growth techniques. These methods include Bridgman growth, the Czochralski method, the floating zone technique, and chemical vapor transport (CVT) [31]. These techniques have traditionally yielded high-quality single crystals of topological materials [32,33,34], revealing valuable insights into their unique topology and band structures. However, there are notable challenges with these traditional single-crystal growth methods, including difficulties in easily integrating them into nanoscale devices with moderate processing temperatures, achieving high conformality, and controlling nucleation and crystallizations on arbitrary interfaces. This is particularly true when producing nanostructured forms of scalable topological materials. Consequently, the potential applications of topological materials in the domain of quantum computation and other cutting-edge technologies have been hindered by a practical gap in controlled fabrication.

**Figure 2:**
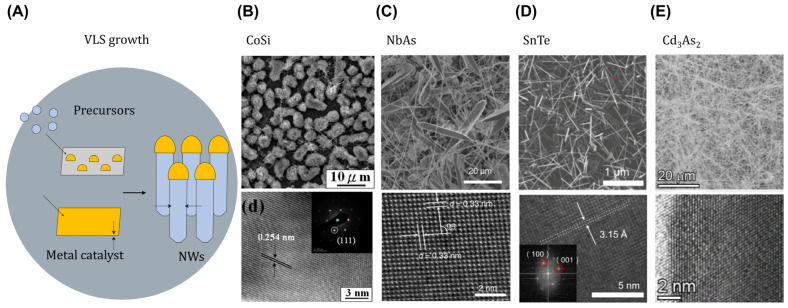
VLS growth utilizing metal catalysts for nanostructured topological materials. (**A**) Schematic illustration of VLS growth. (**B**) Topological chiral cobalt silicide nanowires obtained from self-catalysis process via the vapor–liquid–solid mechanism. (**C**) Weyl semimetal NbAs nanobelts utilizing a gold catalyst. (**D**) Topological crystalline insulator tin telluride grown using Au-Sn-Te alloy nanoparticles. (**E**) Dirac semimetal Cd_3_As_2_ formed by Bi catalytic nanoparticles. (**B**) Adapted and modified with permission [35]. Copyright 2010, IOP Publishing. (**C**) Adapted and modified with permission [38]. Copyright 2023, Wiley-VCH. (**D**) Adapted and modified with permission from [39]. Copyright 2020 American Chemical Society. (**E**) Adapted and modified with permission from [40]. Copyright 2020 American Chemical Society.

To control nanoscale crystallization (Figure 2A), nanostructured topological materials can be confined one-dimensionally (1D) using VLS growth [1]. In VLS growth, metal nanoparticles, often composed of gold, act as catalysts to initiate growth. Source materials in a vapor phase are introduced into the system in a CVD process. When the growth temperature is reached, the metal catalyst enters a liquid state, causing gas phase atoms of the precursor to dissolve into the liquid metal particles. As the concentration of precursor atoms in the liquid metal becomes higher than the thermodynamic solubility limit at the growth temperature, these dissolved atoms aggregate and crystallize at one end of the liquid metal particle. By sustaining a constant supply of source vapor, an equilibrium state is achieved. During this phase, the metal particle expels excess dissolved atoms to uphold the solubility limit concentration, consequently extending the nanowire’s length. 

A distinctive aspect of VLS growth is that the nanowire’s diameter is precisely dictated by the dimensions of the metal nanoparticle, which is essential to determine the diameter-dependent electron scatterings, especially for evaluating low-dissipation interconnects using TSMs. Cobalt silicide (CoSi) nanowires with a maximum current density of 1.6 × 10^8^ A·cm^−2^ in Figure 2B were grown with a 2 nm thick Au catalyst. An Au–Si eutectic alloy was formed at the growth temperature, serving as a Si source [35]. In diameter confined CoSi slabs with 8~40 atomic layers, the calculated resistance area product of the nanoslabs is significantly lower than that of the bulk. However, contrary to theoretical calculations regarding the surface state’s dominant transport of CoSi nanoslabs [36], the resistivity values of VLS-grown 1D CoSi (326 μΩ·cm) and MOCVD-grown 1D Co-Si NWs (~510 μΩ·cm) without any catalyst exhibited markedly higher values compared to that of the bulk crystal (180 μΩ·cm), despite their high surface-to-volume ratio [35,37].

By manipulating the thickness of the gold catalyst layer below 15 nm, Weyl semimetal Niobium arsenide (NbA) nanobelts were grown, as shown in Figure 2C, showing a typical width in the range of hundreds of nanometers and a thickness around ~200 nm. An increase in the initial gold thickness to 40 nm only led to the growth of NbA nanowires. In the case of the NbA nanobelts, there was an anomalous resistivity reduction from 35 μΩ·cm (bulk crystals) to 3 μΩ·cm at room temperature, governed by the surface state’s dominant transport. In the nanobelt configuration, the confinement along the z-direction minimally affected the surface transport properties. However, the transformation into NbA nanowires exposed surface carriers to experience boundary scatterings due to strong in-plane confinement, which led to a degradation in mobility and higher sheet resistance (>100 Ω) of the NbA nanobelts with a thickness of ~100 nm. This phenomenon poses challenges for scaling down towards low-resistivity interconnects based on NbAs [38]. 

The 1D confinement effect may still lead to even richer physics due to the increased contribution from the topological surface states. In the topological crystalline insulator SnTe in Figure 2D, 1D confinement affects the ferroelectric transition temperature, as systemically revealed in in situ cryogenic transmission electron microscopy [39]. Surface defects in the 1D geometry also can be utilized as trap sites for photogenerated electrons in cadmium arsenide (Cd_3_As_2_) nanowires in Figure 2E [40].

**Figure 3:**
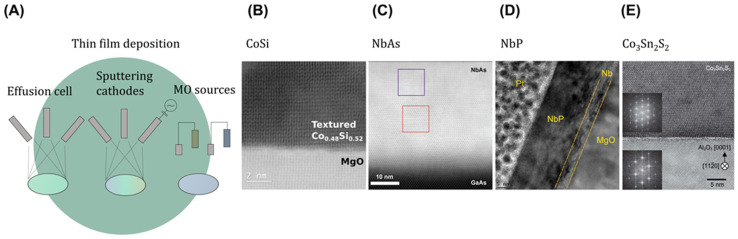
Thin film depositions of Dirac, Weyl, and topological semi metallic nanostructures. (**A**) Schematic of precursor supply units for MBE, co-sputtering, and MOCVD. (**B**) MBE-grown textured CoSi thin films grown on a MgO template. (**C**) NbAs grown on GaA (100) substrates. (**D**) Weyl semimetal NbP thin films grown on a MgO template with a Nb buffer layer. (**E**) Co-sputtered Weyl semimetal Co_3_Sn_2_S_2_ thin films. (**B**) Adapted under the terms of the CC BY-NC-ND 4.0 DEED [47]. (**C**) Adapted under the terms of the CC-BY Creative Commons Attribution 4.0 International license [48]. (**D**) Adapted under the terms of the CC-BY Creative Commons Attribution 4.0 International license [49]. (**E**) Adapted and modified under the terms of the CC-BY Creative Commons Attribution 4.0 International license [52].

Next, two-dimensional (2D) confinement also needs to be considered for utilizing the modification of band structures, in-plane anisotropic transport, and Fermi arc state-dominated electrical conduction with decreasing thickness. To achieve the desired 2D-confined thin films in controlled growth mode using molecular beam epitaxy (MBE), metal–organic chemical vapor deposition (MOCVD), and co-sputtering depositions (Figure 3A), we need to consider two key factors. The first factor involves thermodynamic variables such as Gibbs free energy, equilibrium, and phase diagrams. The second factor encompasses kinetic variables like diffusion rates, enabling preferential diffusion along the a and b axes (in-plane direction) compared to the c axis (vertical direction). Consequently, achieving optimal thin film growth requires a balance of both thermodynamic and kinetic control [41,42]. To host topological surface states in thin films, the ideal Fermi level should inherently be positioned within the bulk bandgap due to the charge balance of these materials. This can be accomplished by minimizing the presence of doping from defects, including vacancies, interstitial atoms, or impurities. Consequently, the Fermi level shifts from its ideal position, moving closer to either the conduction or valence bands. This balance can be effectively achieved through advanced deposition techniques such as MBE, MOCVD, and co-sputtering techniques.

The MOCVD technique includes a wide range of metal–organic precursor flow rates within reactors and is achieved through electrical pressure controllers and mass flow controllers, enabling high-throughput and the mass production of exotic quantum materials [43,44,45]. In MBE, the precise supply of source materials is employed by effusion cells to achieve the controlled evaporation and deposition of thin films. As the precursor material vaporizes, it forms a directed flux of atoms or molecules within the effusion cell in straight lines without colliding with other particles, ultimately allowing complex thin films with controlled heterointerfaces, symmetry, and minimized contaminants under UHV conditions [46]. 

Targeting lower-resistance area products of CoSi thin films for topological semimetal interconnects, which host larger contributions of topologically protected Fermi arc conduction compared to that of the bulk, high-quality textured Co_1−x_Si_x_ was grown in the range of BEOL-compatible 175–400 °C via MBE. However, the sensitivity of MBE-grown Co_1−x_Si_x_ thin films to structural and chemical disorder poses a challenge for the practical implementation of topological metals in nanoscale interconnects and topological devices (Figure 3B,C) [47,48]. Epitaxial 9–70 nm thick NbP thin films revealed topological surface states with a linear dispersion in Figure 3D, along with a Fermi-level shift of −0.2 eV due to Nb vacancies, which helped to stabilize thin films with a larger lattice constant (1%) and P-rich stoichiometry compared to the bulk crystals [49]. These techniques provide tunable growth environments and parameters, allowing precise control over both thermodynamics and kinetics to guide the growth process. 

Additionally, co-sputtering offers several advantages. By applying a high-frequency electromagnetic field to an inert gas in the chamber, the RF source generates plasma. This plasma contains high-energy ions from the gas and, also, from the target materials due to sputtering, causing atoms to be ejected from their surfaces to form thin films. When using multiple targets, it allows the composition of the deposited film to be precisely controlled by adjusting the power applied to each target, making it possible to quickly screen diverse materials with specific compositions [50]. Co-sputtering stands out as a powerful method for precisely adjusting the Fermi level within the band structure compared to traditional bulk crystal synthesis. Notably, the unique properties of TIs [51], topological crystalline insulators, and 3D Dirac/Weyl semimetals become pronounced when the Fermi level aligns with specific points such as 2D Dirac or 3D Dirac/Weyl, ensuring the dominance of nontrivial states. Furthermore, achieving topological superconducting phases by hosting Majorana fermions relies on positioning the Fermi level within the energy gap’s midpoint in topological superconductors. The Weyl semimetal Co_3_Sn_2_S_2_ with the kagome lattice in Figure 3E can induce flat bands near the Fermi level connecting two Weyl cones by electronic correlations, as reported using the co-sputtering technique [52,53].

**Figure 4:**
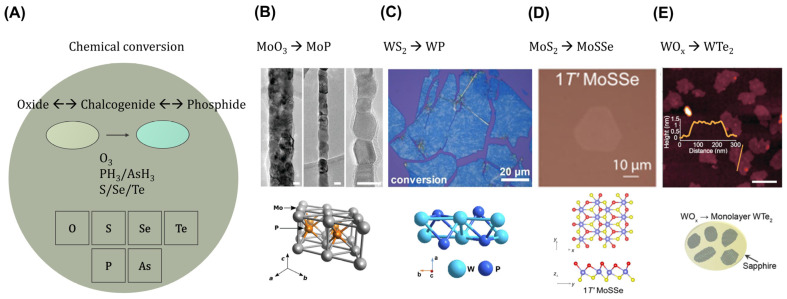
Chemical conversion of nanostructured topological materials. (**A**) Schematic of chemical conversions between oxides, chalcogenides, and phosphides. (**B**) Triple-point metal MoP converted from a 1D MoO_3_ template. (**C**) Topological superconductor WP obtained from the conversion of WS_2_ nanoflakes. (**D**) Topological semiconductor Janus 1T’ MoSSe. (**E**) Monolayer of WTe_2_ converted from ALD-grown WO_x_ thin films. (**B**) Adapted with permission [54]. Copyright 2023, Wiley-VCH. (**C**) Adapted with permission [57]. Copyright 2022, Wiley-VCH. (**D**) Adapted under the terms of the CC-BY Creative Commons Attribution 4.0 International license [59]. (**E**) Adapted under the terms of the CC-BY Creative Commons Attribution 4.0 International license [60].

Chemical conversion, shown in Figure 4A, is a process used to transform materials in the confined dimensions of templates, often involving the conversion of transition metal oxide or chalcogenide (such as sulfides, selenides, and tellurides) compounds into different chalcogenides or phosphides or oxides as well. This transformation is achieved through the replacement of arbitrary host lattice elements by the careful control of temperature; pressure; and gaseous reactants (e.g., ozone, phosphine, arsine, and hydrogen chalcogenide) to ensure a successful substitution of atoms while maintaining the desired crystal structure. During the chemical conversion, the confined environment of the nanocrystal can impact the reaction kinetics and thermodynamics due to surface energy considerations and dimensional confinements. As a result, the chemical conversion process may lead to the formation of unique nanocrystalline structures with exotic properties compared to their bulk equivalents. 

Utilizing phosphine gas, a 1D template of MoO_3_ was successfully converted to low resistivity molybdenum phosphide (MoP) metal lines in Figure 4B and showed superior dimensional scaling with respect to Cu with a TaN barrier, which is not an available phase in the CVD growth of MoP_2_ compounds and the nano-molded metastable Mo_4_P_3_ phase [54,55,56]. A conversion method for WP using 2D chalcogenide templates was also reported, but it resulted in a distorted tungsten phosphide sheet due to the considerable lattice mismatch between WS_2_ and WP, as shown in Figure 4C [57]. By precisely controlling the hydrogen plasma gases, the specific positions of chalcogens can be substituted to realize a highly crystalline Janus monolayer of topological semiconductors (Figure 4D) [58,59]. To enhance our comprehension of chemical conversion, diffusion on particular substrates and the reactivity of transition metals and chalcogens must be carefully considered (Figure 4E) [60].

## 3. Future Perspectives on Topological Nanomaterials

Topological nanomaterials, characterized by their distinctive properties, herald transformative applications across a spectrum of fields, such as the quantum Hall effect, topological insulator behavior, and the Majorana fermions, have significant implications for the advancement of quantum computing. Furthermore, these materials have potential in the development of spintronics and contribute to the innovation in interconnect technologies and sophisticated electronic systems.

**Figure 5:**
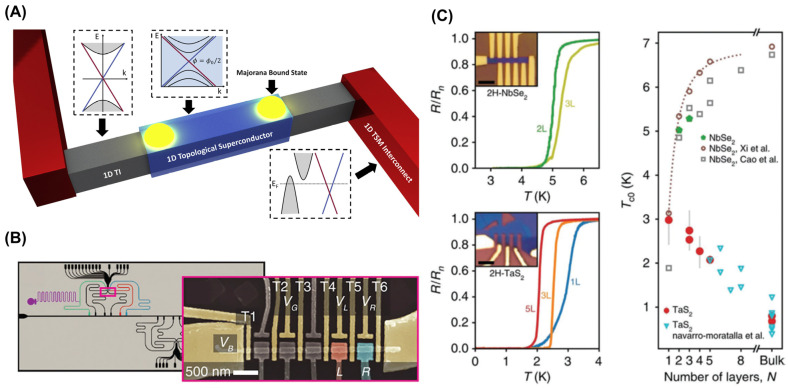
(**A**) Schematic of a hypothetical nanodevice with a topological superconductor (yellow spheres: Majorana-bound states). (**B**) InA multi-QD NW coupled to a microwave resonator. (**C**) Layer-dependent resistivity vs. temperature curves for 2H-NbSe_2_ (**Left Top**) and 2H-TaS_2_ (**Left Bottom**). Layer-dependent critical temperature for NbSe_2_ and TaS_2_ (**Right**). (**A**) Adapted with permission [2]. Copyright 2021, Elsevier. (**B**) Adapted under the terms of the CC-BY Creative Commons Attribution 4.0 International license [75]. (**C**) Adapted under the terms of the CC-BY Creative Commons Attribution 4.0 International license [76].

The foremost potential of topological nanomaterials lies in the prospective realization of topological superconductors, capable of supporting localized Majorana-bound states (MBSs) that are essential for fault-tolerant and scalable topological quantum computing, as depicted in Figure 5A. The progress in superconducting (SC) Josephson junction qubits has been effectively combined with noisy intermediate-scale quantum processors to carry out basic quantum algorithms [61,62]. 

Two-level systems within dielectric tunnel junctions, along with the adjacent dielectric environment, constitute the primary sources of both noise and decoherence in SC qubits [63]. Material and interfacial engineering of both the superconductor and the weak link layer are essential for improving the coherence times of SC qubits. While quantum dot (QD)-based spin qubits have achieved some success, 1D In–V nanowires have emerged as a promising option for topological qubits. These nanowires provide an excellent platform for investigating superconductor–semiconductor hybrid devices, such as gatemon qubits [64] and Andreev spin qubits [65]. In parallel, 2D semiconductors and graphene are gaining attraction for their potential in gatemon qubits [64,66].

Indium antimonide (InSb) and indium arsenide (InA) are garnering significant attention in the field of quantum devices because of their exceptional electron mobility [66], Landé g-factor [67], and spin–orbit coupling (SOC) [68]. In–V nanowires have predominantly been studied for topological systems [69]. The utilization of spin–orbit qubits in these nanowires is promising for next-generation quantum devices. InSb/A nanowires have been used to demonstrate single- [70], double- [71,72], and multi-QD devices [73]. Additionally, a spin–orbit qubit was realized in an InSb NW quantum device [68], and In–V NWs were successfully integrated into superconducting microcavities [71,74,75]. Figure 5B shows an illustration of an InA double QD coupled to a microwave resonator. However, hyperfine decoherence limits the use of In–V NWs for pure QD qubits as planar III–V heterostructures. Given these limitations, the applicability of In–V NWs in practical QD spin qubits appears to be more limited than that of group IV-based 1D nanomaterials. Nonetheless, their prospects in topological and Andreev qubits remain promising.

In parallel, 2D materials offer an advantage and research potential because of their precisely controlled thicknesses and being free of dangling bonds. The potential of 2D superconductors in superconducting qubits has been reported, particularly noting the properties of transition metal dichalcogenide (TMD) superconductors. For instance, NbSe_2_ exhibits a significant critical field, attributable to Ising SOC [76,77,78], and monolayer FeSe, epitaxially grown on oxide substrates, has been demonstrated to be a high critical temperature superconductor [79,80]. Ising spin–orbit protection allows for the significant Ising SOC in TMDs, resulting in the ability to enable substantial in-plane critical magnetic fields [81]. Superconductivity extending to the monolayer has been validated in NbSe_2_ and TaS_2_, with size effects on the critical temperature (T_C_), which vary distinctly between materials. T_C_ increases as the thickness is reduced in TaS_2_ but decreases in NbSe_2_, as detailed in Figure 5C [76]. Furthermore, all 2D Josephson junctions with highly transparent interfaces have been achieved [82,83] and offer a promising avenue. However, challenges persist in the pursuit of growing 2D materials at a wafer scale with high crystallinity and minimal defects, a task relatively more manageable in the established wafer-scale deposition processes for Al, Nb, and Al oxide for SC qubits.

Although obstacles have been encountered in the study of one-dimensional topological superconductors and the detection of Majorana-bound states (MBSs), there is a renewed interest in another promising application involving topological nanomaterials. Specifically, the utilization of topological surface states for low-dissipation interconnects has garnered attention because of its potential to address existing challenges in interconnect technology [2,84].

**Figure 6:**
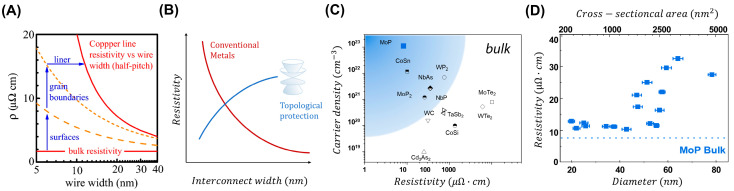
(**A**) The effective resistivity (ρ) of copper (Cu) interconnect lines with respect to wire width. (**B**) The schematic illustrates the resistivity of interconnect widths for conventional metals and materials with topologically protected surfaces. (**C**) Comparative plot of the carrier density and resistivity values of TSM as reported in the literature. (**D**) The room temperature resistivity of molybdenum phosphide nanowires on various nanowire diameters. (**A**) Adapted with permission [84]. Copyright 2021, Springer Nature. (**C**,**D**) Adapted with permission [54]. Copyright 2023, Wiley-VCH.

The escalating resistance of copper interconnects poses a significant obstacle for ongoing reduction beyond the 7 nm node. This challenge is further compounded by the need for a liner and barrier layer to protect the copper interconnects. Currently, research is focusing on alternative metals, such as ruthenium (Ru) and cobalt (Co) [85,86], as potential replacements for copper (Cu) without the requirement of a barrier layer. Notably, these alternatives still exhibit enhanced surface scattering effects at the nanoscale, as depicted in Figure 6A [87].

The discovery of TIs has generated significant enthusiasm in their potential as interconnect materials, driven by their theoretically predicted minimal dissipation. This interest stems from the theoretical prediction that TIs possess topological surface states characterized by both spin polarization and a lack of scattering. Such characteristics are anchored in theoretical forecasts of dramatically reduced resistivity with dimensional scaling, as shown in Figure 6B. However, recent findings have indicated that TIs may not be suitable for interconnects [2,8,84]. For instance, the mobility of Bi_2_Se_3_ nanoribbons is notably reduced at room temperature due to the influence of electron–phonon interactions. These interactions affect both the surface and bulk state’s mobility [88]. Additionally, interconnects necessitate a sufficient carrier density that is reasonably high and a resistivity that is low, comparable to that of copper. Most TIs do not meet these requirements. 

Recently, there has been a revived interest in examining topological semimetals (TSMs) as interconnect materials. TSMs, in contrast to TIs, offer a significant bulk carrier alongside the topological surface states that reside at the Fermi level. Several transition metal dichalcogenides (TMDs), such as MoP, molybdenum ditelluride (MoTe_2_), tungsten carbide (WC), and tungsten diphosphide (WP_2_), have exhibited promising characteristics such as high carrier densities and low resistivity values, as shown in Figure 6C [34,89,90,91].

These properties reignite the initial potential of topological materials as interconnects with minimal dissipation in extremely small dimensions. Several recent studies in the field of nanostructured TSMs have demonstrated promising results. For example, the resistivity of Weyl semimetal NbA nanoribbons (3 μΩ·cm) is dramatically lower compared to the bulk value (35 μΩ·cm), since transport is predominantly via topological surface states [38]. Likewise, a recent theoretical analysis suggests that the transport properties of CoSi should be primarily governed by topological surface states. This could potentially lead to a lower line resistance than Cu at the 5 nm technology node [36]. Moreover, MoP topological metal nanowires were demonstrated as a breakthrough material for interconnect applications, exhibiting superior dimensional scaling of resistivity compared to effective Cu (Cu with a TaN liner) and Ru, as shown in Figure 6D [54]. 

Spintronic devices based on topological materials have recently attracted great attention. For example, TIs have been extensively explored for spin transport detection due to their unique conduction states, characterized by spin momentum locking [6,92]. Applying a direct current generates an electric field (E) that causes Fermi surface shifts, resulting in the acquisition of a net momentum (ke). The outcome of this process is a spin-polarized current with polarization (S). This is a result of the helical spin nature [93]. Generally, a spin current in TIs is detected using ferromagnetic (FM) tunneling contacts. In this setup, the measured voltage is influenced by the alignment of the induced spin polarization and the FM magnetization (M) [94].

Numerous TI materials, such as Bi_2_Se_3_ [93,95], (Bi_1–x_Sb_x_)_2_Te_3_ [96], and Bi_2_Te_2_Se [17], have demonstrated the electrical detection of current-induced spin polarization. Notably, the spin momentum locking characteristic in TIs is particularly beneficial for the process of magnetization switching through spin–orbit torque. Many researchers have noted that the net spin accumulation on the surface can efficiently control the magnetization [97,98,99,100,101,102,103,104]. 

Additionally, surface states with a large Rashba splitting can also result in spin polarization under an electric current. The current induced spin polarization, which arises from the surface band bending effect, exhibits a sign opposite to that of the surface states [94]. In contrast to the Dirac surface states, the 2D Rashba states show a parabolic energy dispersion and can have their spin helicity electrically modulated to the opposite direction [105], unlike the topological surface states.

**Figure 7:**
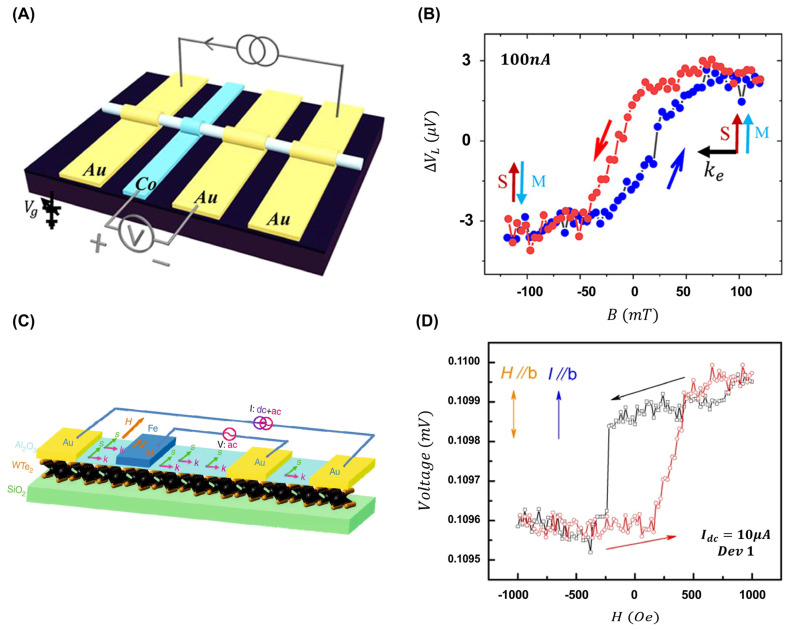
(**A**) Schematic image of measuring the local spin using a Cd_3_As_2_ nanowire device. (**B**) Magnetic hysteresis loops were measured using a direct current in the local region. (**C**) A schematic image of a WTe_2_/Al_2_O_3_/Fe tunnel junction device. (**D**) The voltage across two inner electrodes of the tunnel junction is measured in relation to the magnetic field within the plane (T = 2 K). (**A**,**B**) Adapted with permission [109]. Copyright 2020, American Physical Society. (**C**,**D**) Adapted under the terms of the CC-BY Creative Commons Attribution 4.0 International license [110].

The Fermi arcs of TSMs have been proposed to exhibit a spin momentum locking characteristic, wherein the spin is locked in-plane. This property holds potential for current-induced spin polarization and practical spintronic applications [7,106,107,108]. For example, a local transport measurement setup on a Dirac semimetal Cd_3_As_2_ nanowire demonstrates this principle, as shown in Figure 7A [109]. The setup involves applying a DC current between outer gold electrodes and measuring the potential difference between the inner electrodes. The application of a bias current establishes a net in-plane spin (S), influencing the measured voltage based on its alignment with the magnetization (M). 

A charge current would induce a spin-polarized current, due to the helical spin nature. A net in-plane spin S is established with a bias current +100 nA, as shown in Figure 7B. When the spin S aligns with the M, a significant response appears in contrast to the reduced voltage observed under nonalignment conditions. Consequently, modulating the in-plane magnetic field near B = 0 T would lead to a hysteretic loop. 

To elucidate the spin signal originating from surface states, the contribution of bulk states has been eliminated in the local measurement. Reversing the bias current causes the surface spin orientation to produce an inverse hysteretic loop, owing to spin momentum locking. Additionally, the spin transport of topological surface states can be observed through a nonlocal measurement when a DC current is applied across adjacent electrodes, and the potential difference is measured between Co and gold electrodes. These nonlocal measurements can significantly decrease the bulk contribution and effectively separate the surface spin signal. The observation of a hysteretic loop indicates the topological protection and the inherent robustness of the surface states against disorders and defects. A similar pattern is observed in the Weyl semimetal WTe_2_, affirming the robustness and topological protection of these surface states against disorders and defects (Figure 7C,D) [110].

## 4. Challenges

Realizing the full potential of topological nanomaterials in advanced electronics requires overcoming significant challenges and furthering innovative advances. Key among these challenges is the synthesis of robust nanoscale topological materials, particularly superconductors, which are crucial for the development of error-resistant topological quantum computers and other advanced electronic devices. So far, the most notable studies to date have utilized semiconducting nanowires that possess a strong spin–orbit coupling [111]. These are typically grown using sophisticated techniques like molecular beam epitaxy or MOCVD with superconductivity induced by superconducting metals. However, the synthesis and transport of these materials at the nanoscale present unique challenges.

To enhance the synthesis of topological nanomaterials, it is crucial to achieve high-throughput and precise control over their morphology, dimensions, crystallinity, and doping. While CVD can produce nanostructures with high crystallinity, it lacks precise control over the dimensions and morphology. A promising approach is the top-down fabrication method, which uses conventional lithography techniques and etching to fabricate nanowires by patterning thin films of topological materials. Nevertheless, this method can introduce surface roughness, potentially impairing the transport properties.

Recent advances in nanofabrication have introduced a thermomechanical nano-molding (TMNM) of ordered phases. A prime example of TMNM’s efficacy is its application in the fabrication of Mo_4_P_3_ nanowires using a bulk MoP feedstock [56]. This process has successfully yielded single-crystal 1D nanowires of Mo_4_P_3_, marking the first instance of achieving these materials. This method offers a top-down approach without the typical defects induced by lithography, allowing for better control over the morphology and facilitating high-throughput screening. Additionally, leveraging data-driven and calculation-based screening techniques can accelerate the synthesis process. These techniques enable the prediction and selection of topological materials suitable for specific applications.

While significant progress has been made in the synthesis of topological nanostructures, there are still several challenges that need to be addressed. Imperfections or defects in the crystal lattice can affect the electronic properties and performance of the materials. Finding ways to minimize defects during the synthesis process or developing post-synthesis treatments to improve the crystal quality could lead to significant advancements. Precise morphological control is another challenge, as the shape, size, and arrangement of nanostructures influence their electronic properties and functionality. Even though recent studies have explored controlling the size and crystallinity through the nanoconfinement effect in conversion techniques [54,55,112,113], developing methods to control and manipulate the morphology of topological nanostructures could open up new possibilities for their utilization in electronic devices. Future research should focus on addressing these challenges and exploring innovative approaches to fabricating topological nanostructures with enhanced crystal quality and precise morphological control.

## 5. Conclusions

In summary, the field of topological nanomaterials is advancing rapidly, showcasing immense potential. However, significant challenges still exist, particularly in the synthesis and transport of topological materials at the nanoscale. Addressing these unique challenges is crucial for further progress. Despite these obstacles, ongoing research and technological advances are constantly enhancing synthesis techniques, thereby enriching our understanding of topological nanomaterials. To fully realize the potential of these materials in cutting-edge applications such as quantum computing, spintronics, interconnect, and advanced technologies, a concerted effort combining both theoretical and experimental research is essential. This comprehensive approach is key to overcoming the existing challenges and unlocking the vast possibilities offered by topological nanomaterials.

## Data Availability

No new data were created or analyzed in this study. Data sharing is not applicable to this article.
